# Periocular necrotizing fasciitis

**DOI:** 10.3389/fopht.2026.1758717

**Published:** 2026-01-26

**Authors:** Zaynab Somani, Hyun Jun Kim, Andrew Harrison, Ali Mokhtarzadeh

**Affiliations:** 1 University of Minnesota Medical School, Minneapolis, MN, United States; 2 Department of Ophthalmology and Visual Neurosciences, University of Minnesota, Minneapolis, MN, United States; 3 Department of Otolaryngology and Head and Neck Surgery, University of Minnesota, Minneapolis, MN, United States

**Keywords:** eyelid cellulitis, eyelid necrotizing fasciitis, necrotizing fasciitis, orbital cellulites, orbital infections, orbital necrotizing fasciitis

## Abstract

Necrotizing fasciitis is a rapidly progressive infection commonly associated with group A *Streptococci* and other toxin-producing bacteria that leads to microvascular thrombosis, tissue ischemia, and fascial plane spread. Although the periorbital region’s rich vascularity and natural anatomic barriers help limit deep extension and systemic spread, periorbital necrotizing fasciitis (PNF) can cause septicemia, systemic toxicity, and death. Early diagnosis is critical but remains challenging; distinguishing PNF from inflammatory mimics such as Sweet’s syndrome is critical given their opposing interventions. Surgical debridement along with intravenous antibiotics remain the cornerstone of treatment. However, considering the significant morbidity from vision loss and disfigurement, the decision to pursue early surgical debridement versus conservative management remains an area of active debate. Emerging blood biomarkers may aid diagnosis, and adjunctive therapies, including hypochlorous irrigation, show promise in improving outcomes of PNF while minimizing treatment-associated morbidities. This mini-review synthesizes current treatment evidence for PNF, integrating findings from PNF-focused literature and studies of generalized-NF at other anatomical sites. While principles utilized in the treatment of generalized NF may be applicable to PNF, exact statistics may vary by anatomic site.

## Introduction

Necrotizing fasciitis (NF) is life-threatening bacterial infection characterized by extensive necrosis of superficial fascia (1) with rapid progression along subcutaneous soft tissue planes. The widespread bacterial load of this infection, often caused by group A *β*-hemolytic *Streptococci (GABHS), Staphylococcus Aureus*, the *Clostridium* species, *Vibrio vulnifuncus*, and other toxin-producing bacteria may result in septic shock (1). Although NF most frequently involves the groin, abdomen, and lower extremities, it can rarely affect the head and neck, including the periorbital region. Despite the robust vascular supply, periorbital necrotizing fasciitis (PNF) carries a high risk of vision loss, systemic complications, and mortality due to potential extension to the cervical viscera and thoracic cavity (2). Prompt diagnosis and treatment are critical to improving visual and survival outcomes. This review encompasses risk factors, pathogenesis, clinical presentation, diagnostic features, and management of PNF cases over the past 30 years.

## Epidemiology

While comprehensive epidemiologic data for all bacterial causes of NF are limited, life-threatening *GABHS* infections, including NF, toxic shock syndrome, and bacteremia, occur at an incidence of 3 per 100,000 individuals annually in the United States (3). NF itself remains a rare but rapidly progressive infection, with an estimated incidence of 0.4 per 100,000 individuals annually in the United States, corresponding to 10,000 cases annually (4). Approximately 10% of NF cases involve the head and neck region, with orbital and periorbital involvement comprising a small subset. One retrospective review identified 58 well-documented case records of PNF over a fifty-year period (5). PNF occurs predominately in adults, with a median age of 46.3 years (6) with incidence increasing markedly after age 80, and mortality increasing after age 50 (7).

Risk factors for NF include diabetes mellitus, chronic renal failure, cardiovascular disease, peripheral vascular disease, rheumatologic disease, drug misuse, alcoholism, malignancy, and immunocompromise (5, 6, 8–11). Disease progression to shock and multi-organ failure has been associated with unidentified body punctures, pressure ulcers in immobilized patients, and delayed medical treatment (12).

The most common precipitating events for PNF are penetrating or surgical traumas, such as blepharoplasty, dacryocystorhinostomy (6), and minor traumas including insect bites (6). Adjacent infections from dacryocystitis, sinusitis, pneumonia, and parotid gland infections have also been implicated (6).

Notably, up to 52% of patients with NF have no identifiable comorbidities or predisposing factors (8, 9, 13).

## Pathogenesis

NF derives its title from the rapid spread of necrosis through fascial planes. Based on microbiologic etiology agents, NF is classified into four types (10). Type I is polymicrobial, typically involving *Streptococcus* species, *Klebsiella* species, *S. aureus*, and *E. coli.* Type II is monomicrobial and most commonly caused by *GABHS* with or without concurrent *S. aureus*. Type III, albeit rare, is associated with marine Gram-negative pathogens including *Vibrio vulnifuncus* and *Clostridium* species. Type IV is fungi in origin, most often involving *Apophysomyces* and *Aspergillus*, and is associated with uncontrolled diabetes (10). Types II, III, and IV have been documented in PNF, with *GABHS* and *S. aureus* representing the most common causative organisms (10).

Periorbital involvement is uncommon due to its distinct natural anatomic barriers. The orbital septum, orbicularis oculi muscle, firm dermal attachments at the nasojugal and malar folds, and a robust vascular supply limit bacterial penetration into deeper fascial layers (10) and reduce the risk of superficial infection.

As such, mechanical damage to the skin as an entry point for inoculation is the most frequent instigator of PNF. After the dermal and muscular barriers are compromised, principal pathogens produce exotoxins driving necrosis and their spread into the orbital apex, throat, and systemic circulation (10). In Type II *GABHS*-predominant NF, superantigens including streptococcal pyrogenic exotoxins (Spe) A and C, in addition to Staphylococcal TSST-1 and enterotoxins (11), induce widespread T-cell activation while bypassing antigen specificity. Simultaneously, the streptococcal protease and virulence factors blunt the neutrophilic and phagocytic response needed for an effective antibacterial response. The resulting pro-inflammatory cytokine surge results in endothelial dysfunction and platelet aggregation (10). Resulting microvasculature thrombosis restricts immune cell trafficking and antibiotic penetration, facilitating horizontal infectious spread along fascial planes (14), ultimately leading to further ischemia, bullae formation, ulceration, necrosis (15), and toxin release. In Type I polymicrobial NF, Gram-negative organisms produce endotoxins and LPS-mediated inflammation, while Type III pathogens, including *Vibrio vulnificus* and *Clostridium* species, generate cytolysins and lecithinase-alpha toxins, yielding myonecrosis, hemolysis, and vascular injury (16, 17). Bacterial hyaluronidase and collagenase can amplify the lateral extension of infection.

## History, signs, and diagnosis

Early NF frequently follows small cutaneous insults such as insect bites or pressure ulcers. These are commonly misdiagnosed as cellulitis (12), resulting in diagnostic delays and progression to critical necrosis spreading to distal limbs (10).

Patients typically present with tense, erythematous periorbital skin (18), pain disproportionate to exam, and rapidly progressive edema that may cross fascial planes despite intact overlying skin (5). Systemic symptoms such as high fever, rigor, and sweating are common. Characteristic signs of PNF include bullae, violaceous-cyanotic discoloration with irregular erythematous borders, and gangrene that develop within 24 hours. Disease progression is rapid with frank cutaneous necrosis, anesthesia due cutaneous nerve destruction, and crepitus developing in 4–5 days (6). Neutropenic patients may lack classic such inflammatory findings (6) necessitating higher diagnostic suspicion. Systemic toxicity-including fever, hypotension, hypothermia, and electrolyte derangements-is frequent (12). In contrast to NF affecting other regions, PNF frequently demonstrates bilateral involvement due to low resistance of subcutaneous tissue across the nasal bridge, facilitating infectious spread in up to 45% of cases (10).

Evaluation of PNF is challenging due to a number of diagnostic pitfalls, including orbital cellulitis (12), zoster, erysipelas, and angioedema (10). As a result such, early diagnosis is reportedly missed in 85 to 100 percent of cases (19). Diagnosis remains largely clinical but is supported by evidence of systemic inflammation, including elevated CRP (10, 20), leukocytosis (6, 10, 21), and increased procalcitonin levels (20). Among these, leukocytosis has been identified as a particularly important indicator and may help differentiate NF from other soft-tissue infections (21) with sensitivities and specificities exceeding 90%. Routine laboratory parameters are incorporated into the Laboratory Risk Indicator for Necrotizing Fasciitis (LRINEC) score, which aids in early detection of NF, particularly in clinically equivocal presentations. A LRINEC score of ≥6 should heighten clinical suspicion for NF (21). Readily available online calculators may serve as an adjunct to bedside risk stratification.

CT and MRI imaging may further support diagnosis by revealing fascial thickening, soft tissue gas, or extension into the orbitofacial compartments, helping distinguish NF from cellulitis or myonecrosis (10, 22). While MRI is superior in localizing the initial infection site, a PNF case report found that CT may sufficiently guide surgical intervention (23). Aerobic and anerobic wound cultures are initiated prior to the administration of narrowed antibiotic therapy, and typically isolate polymicrobial flora including *GABHS* and *S. aureus*. Blood cultures are low in sensitivity and not well supported in literature (6). Frozen section biopsy can confirm facial necrosis, revealing dense collections of gram-positive cocci (6). Emerging hematologic indices such as the neutrophil-to-lymphocyte and neutrophil-to-platelet ratios show promise in differentiating PNF from orbital cellulitis (24).

Necrotizing Sweet’ syndrome, a rare neutrophilic dermatosis, may mimic NF clinically and radiographically. It’s shared tender, erythematous presentation but opposing treatment presents a crucial diagnostic pitfall, making histopathologic evaluation (showcasing neutrophilic infiltrate without microorganisms) critical for distinction (25).

## Treatment

PNF requires urgent intervention to reduce mortality and preserve ocular function. Prompt initiation of empiric broad-spectrum antibiotics is critical. Standard empiric PNF therapy should include β-lactams for streptococcal and gram-negative coverage, clindamycin to suppress toxin synthesis (5), and an aminoglycoside or metronidazole for polymicrobial or anaerobic coverage. Clindamycin should not be used as monotherapy due resistance concerns (6). Once culture data are available, therapy is narrowed accordingly. PNF’s thrombotic character impedes antibiotic penetration to the site (6), supporting the frequent need for surgical debridement of the affected tissue in conjunction with antibiotic therapy.

Surgical debridement remains the cornerstone of PNF management. Early removal of necrotic tissue reduces bacterial load, toxin-mediated tissue injury, and tissue ischemia, while retaining as much healthy skin and orbital tissue as possible (5). However, the rich periocular vascular supply and functional morbidity with tissue loss allows clinicians to exercise clinical judgement. Some series advocate extensive debridement of subcutaneous tissue until vascularized tissue is encountered, with conservative excision of overlying skin (5). Other PNF series (26, 27) support a tissue-sparing approach, particularly in patients with stable or improving inflammatory markers, involving selective removal of eschars and frank necrosis while allowing for granulation of junctional tissue (26, 27). These findings suggest that aggressive early excision may not be universally required in PNF, in contrast to generalized NF, where an average of 3 debridements is often required for complete source control (12). Exenteration remains controversial, but may rarely be indicated when necrotizing inflammation spreads into the orbit (5).

Topical hypochlorous acid (0.01% HOCl) has emerged as a promising adjunct in NF for wound irrigation due to its anti-microbial, antitoxin, and antibiofilm activity with minimal host cytotoxicity (11, 27). Flow-through delivery via vacuum-assisted closure (VAC) systems used in generalized-NF may be applicable for complex periocular cases (11). Additional adjunctive therapies, including intravenous immunoglobulin (IVIG) to neutralize toxin activity and hyperbaric oxygen to limit tissue ischemia, have been reported though robust clinical data supporting their routine use is lacking (6, 10).

## Management

Following debridement, wound care is essential for recovery. Contemporary protocols increasingly employ negative pressure wound therapy (NPWT), VAC systems, or vacuum sealing drainage (VSD) dressings to control wound exudate and promote granulation. A case series of generalized-NF utilized sized V.A.C. GranuFoam, V.A.C. VeraT.R.A.C, or V.A.C. VeraFlo dressings with flow-through HOCl irrigation (delivered through inflow tubes constructed from intravenous line extensions) sealed with Stomadhesive Paste and adhesive drape (11). Another retrospective analysis of generalized-NF described postoperative VSD with twice-daily irrigation containing insulin, lidocaine, and recombinant acidic fibroblast growth factor in saline solution to stimulate granulation (12).

PNF wound management emphasizes repeated debridement until all necrotic tissue is removed followed by irrigation with normal saline (7, 10). Regular bedside dressing changes with non-adherent, moisture-retentive materials should be performed. The extent of tissue loss informs reconstruction. Delayed split- and full-thickness skin grafts are effective in management of postoperative lagophthalmos and ectropion. Large defects may require fasciocutaneous free flaps to restore eyelid function (7).

## Association with COVID-19

Recent reports have described an increased incidence of head and neck NF during the COVID-19 pandemic, suggesting a potential association. One generalized-NF case series described higher post-pandemic case counts, an increased need for debridement, and 12.5% increased rate of mortality (28). Proposed explanations include pandemic-related delays in care and worsening glycemic control among diabetics as well as COVID-associated endothelial injury, hypercoagulability, and immune dysregulation (29).

## Outcomes of periocular necrotizing fasciitis

PNF carries a more favorable prognosis than NF of other anatomic sites, with a reported mortality of 6-15% (7, 10, 18), compared to 20-30% for NF involving the head, neck, or extremity (7). Mortality is associated with delayed diagnosis (10), extension into cervical and thoracic compartments, and toxic shock syndrome (5). Septic shock has been identified as the strongest predictor of mortality, and was associated with facial involvement, visual impairment, and (albeit rare) Type 1 infections (6).

Rates of orbital exenteration vary widely. One retrospective study documented exenteration in 4 of 7 cases (57%) and 2 of 58 cases (3%) (5), while a separate review reported exenteration in 7 of 94 cases (7.4%) (6). Although central nervous system complications have not been quantified in periorbital NF specifically, orbital necrotizing infections (a natural progression of PNF) have been associated with intracranial spread (27, 29, 30), including meningitis, cavernous sinus thrombosis, and brain abscess, with reported mortality rates up to 14% (29).

Visual morbidity remains a chief cause of long-term disability. One PNF series reported blindness in 5 of 7 patients (71.4%) despite early surgical intervention, whereas another noted loss of vision in 13 of 94 cases (13.8%) (6). In addition to visual loss, soft tissue defects result in functional and cosmetic sequelae (6, 10).

## Discussion

PNF remains a diagnostic and therapeutic challenge due to its subtle early manifestations, rapid progression, and potential visual morbidity. This review synthesizes evidence from primary clinical studies, systematic reviews, and narrative reviews to provide an updated perspective on contemporary diagnostic and management principles for PNF. **Table 1** represents original clinical reports of periorbital or generalized-NF that provide anatomic characterization of infection sites, medical or surgical interventions, and documented outcomes.

**Table 1:** Summary of primary literature on periorbital and generalized necrotizing fasciitis.

Study	Study type	Site(s) involved	Intervention(s)	Outcome(s)
Kronish et al., 1991 (2)	Case report (n=1) and literature review	Periorbital	Early surgical debridement, reconstruction, broad-spectrum IV antibiotics, and adjunct hyperbaric oxygen therapy	Levator recovery with mild ptosis; no mortality.
Elner et al., 2006 (5)	Retrospective review (n=7)	Periorbital, cheek, neck, chest, extremities	Clinically aggressive surgical subcutaneous debridement with histopathologic evidence	5 patients suffered loss of vision, 1 death due to septic shock.
Yu et al., 2022 (12)	Retrospective analysis (n=35)	Lower extremities, sacrococcygeal area, chest	Early and aggressive surgical debridement, VSD ± medium-thickness skin graft	All patients survived (n=35), 22 underwent repeat debridement, 30-day average hospitalization, no reoccurrence observed.*
Hadizamani et al., 2023 (10)	Case report (n=1)	Periorbital	5 days of daily surgical debridement, outcomes prompting secondary surgical correction	Recovery without need for skin grafts with ptosis, dermatochalasis, inferior lid ectropium (corrected).
Oliver-Gutierrez et al., 2024 (13)	Case series (n=9)	Periorbital	Surgical debridement, broad-spectrum IV antibiotics	2 cases of toxic shock, with no fatalities. One exenteration. 5 cases with residual sequelae (ptosis, lagophthalmos, exposure keratopathy, etc.). Final BCVA in preserved eyes 0.1–1.0.
Saldana et al., 2010 (23)	Case report (n=5)	Periorbital	CT-guided surgical debridement	No fatalities. 4 patients had minimal surgical debridement to the surface muscle. 4 patients underwent delayed reconstruction.
Hopkins et al., 2025 (31)	Case series (n=5)	Periorbital	Early IV antibiotics and urgent surgical debridement	All patients underwent up to 4 debridements. No fatalities. 2 cases of post-septal involvement. 3 cases of orbital compartment syndrome, with two resulting in VA of NPL. 4 cases of lagophthalmos, 2 cases of exposure keratopathy, 1 case of ectropion. Use of LRINEC unreliable.
Tambe et al., 2012 (32)	Case series (n=11)	Periorbital, face, neck, chest	Broad-spectrum IV antibiotics and surgical debridement	One fatality due to systemic shock and multiorgan failure. One case of lost site in the affected eye due to CRAO. Remaining 9 cases preserved vision with acceptable functional and cosmetic results. LRINEC score did not correlate with PNF severity.
Rajak et al., 2016 (27)	Case series (n=29)	Periorbital, face, neck, scalp.	IV antibiotics with cautious observation, surgical debridement	Up to 5 tissue debridements to control disease in 23 patients. One fatality prior to debridement. One case of intracranial spread. 6 cases of systemic shock. Visual loss occurred in 4 eyes of 4 patients.
Mutamba et al. (2013) (26)	Case series (n=3)	Periorbital	IV or oral antibiotics, delayed “elective” debridement	Debridement underwent in all 3 cases. All 3 cases underwent reconstructive skin grafting. All achieved satisfactory cosmetic result. One case of lacrimal symptoms due to static eye. One case’s VA noted to improve from HM at presentation to 6/9.
Hu et al., 2008 (18)	Case report (n=1)	Periorbital	High dose IV antibiotics, bedside debridement	Post-antibiotics: No necrotic progression, gradual improvement of affected area. Post-debridement: Return of EOMs, VA at HM.
Işik et al., 2025 (20)	Case report (n=1)	Periorbital	Broad-spectrum IV antibiotics, IVIG, surgical drainage/fasciotomy, and hypochlorous acid irrigation	Resolved without sequelae.
Crew et al., 2016 (11)	Case series (n=6)	Extremities, trunk, genitalia	Flow-through 0.01% HCl via NPWT as adjunct to surgical debridement and antibiotics ± hyperbaric oxygen therapy	All patients achieved infection resolution and wound healing within 2–8 weeks; no mortality or recurrence reported; HOCl with NPWT facilitated rapid recovery and improved cosmesis.

NPWT, negative pressure wound therapy; BCVA, best corrected visual acuity; VSD, vacuum sealing drainage; EOM, extraocular movements; VA, visual acuity; NPL, no perception of light; HM, hand motion; IVIG, IV immunoglobulin

*Among re-examined patients at one year follow-up

Early recognition of PNF is critical, as prompt surgical debridement remains as the foundation of management. However, PNF may progress rapidly before cutaneous necrosis becomes apparent, and symptoms such as disproportionate pain, systemic toxicity, or rapidly expanding edema warrant urgent imaging to confirm fascial involvement (6, 22). CT or MRI imaging is also indicated to differentiate PNF from pre-septal, orbital cellulitis, myonecrosis, and other masquerades (6). Key findings on CT include soft tissue gas, superficial fascial thickening or nonenhancement (6), fluid tracking along fascial planes (5), loss of normal fat planes, and edema extending beyond the region of visible skin changes (6). One PNF case report demonstrated CT alone was sufficient to guide surgical debridement (21). MRI is more sensitive for early disease and superior delineation of infection extent (22). Characteristic findings include T2-weighted hyperintensity of the deep fascia and post-contrast T1-weighted fascial nonenhancement suggestive of necrosis, muscle edema, or myonecrosis.

While the LRINEC score may support early risk stratification in NF, its applicability in PN is limited. The score has not been found to correlate with PNF severity (31, 32) and may not account for neutropenic or immunocompromised patients. Accordingly, LRINEC should be interpreted only as an adjunctive tool, and low scores should not delay surgical intervention when clinical suspicion remains high.

The timing and extent of debridement remain among the most debated aspects of PNF management. Early and aggressive debridement improves source control and reduces mortality, but also carries substantial functional and cosmetic morbidity (6, 10). The periorbital region’s rich vascularity simultaneously protects against disease progression and complicates management. Extensive arterial anastomoses of the superior and inferior superficial arcades provide robust perfusion that enhance immune cell trafficking essential for localized control of infection and may explain the reported success of conservative management with intravenous antibiotics in select patients with involvement limited to the eyelids without systemic comprimise (5, 6, 18). Several case series support selective debridement strategies that preserve junctional tissue adjacent to viable skin, allowing auto-demarcation and granulation in clinically stable patients. (26, 27) Thus, management often involves a nuanced balance between aggressive source control and tissue preservation, combining surgery and antimicrobial therapy based on disease severity and progression. Nevertheless, conservative therapy requires a high index of suspicion (6) and close monitoring.

The same vascularity that may protect against progression can also mask disease severity. Viable-appearing skin may overlie necrotic subcutaneous tissue while local microvascular thrombosis limits antibiotic penetration. Clinical indicators such as disproportionate pain, rapid edematous progression, cyanotic-violaceous discoloration, cutaneous anesthesia, bullae necrosis, and systemic toxicity should heighten concern for advancing necrosis (6). Early surgical debridement is indicated with evidence of systemic toxicity and deep fascial or orbital involvement (including extension beyond the orbital septum and loss of tissue plane integrity), rapid progression, or gaseous formation on imaging (5). These features signal deeper necrosis and prompt urgent surgical consultation, as delays beyond 24 hours can markedly worsen prognosis and tissue destruction (6). Consequently, debridement remains a cornerstone of therapy and is often repeated until viable tissue margins are achieved (5, 12). In contrast to NF at other anatomical sites, PNF may permit a less aggressive surgical approach due to robust regional vascularity and the high functional cost of tissue loss.

Adjunctive treatments, such as topical HOCl irrigation (11, 33) or NPWT (11, 34) may enhance local infection control while minimizing surgical morbidity. A generalized-NF case series supported the use 0.01% pure HOCl applied through a flow-through NPWT at high frequency every four to six hours, with five minutes of treatment time per cycle (11). A pediatric PNF case report irrigated and performed drainage changes every two hours (21). This regimen is justified by HOCl’s rapid consumption of protein-rich necrotic tissue and neutralization of streptococcal superantigens, staphylococcal cytotoxins, and anaerobic proteases (33). Therapy is continued until acute inflammation resolves, generally over one to three weeks (11, 20). As the periocular region is difficult to seal and more vulnerable to pressure-related injury, adapting this HOCl-NPWT technique to PNF would require modified ophthalmic interfaces and further study. In contrast, general wound care protocols, such as diabetic-venous foot ulcers and postoperative wounds, employ once-daily HOCl with treatment times of 15 to 20 minutes (33), reflecting lower rates of toxin production and necrotic load.

If standard PNF treatments fail or clinical features are atypical, alternative diagnoses must be considered. Type IV fungal NF should be suspected in diabetic or immunocompromised patients. Sweet’s syndrome (SS) represents a critical diagnostic pitfall. SS presents with painful, erythematous plaques or nodules, fever, and neutrophilia and its necrotizing variant can closely mimic PNF (25). Histopathology distinguishes SS by demonstrating dense neutrophilic infiltrate without microorganisms or vasculitis (25, 35). Misdiagnosis has grave consequences: PNF typically requires urgent surgical debridement and antibiotics, whereas SS responds to systemic corticosteroids. Inappropriate debridement in SS results in pathergy (35). Accordingly, in addition to early use of imaging when uncertainty persists, prompt biopsy is essential.

Current research gaps remain. These include advanced PNF-NPWT wound dressings and standardized criteria to guide conservative versus surgical management (5, 10). Robust comparative data on adjunctive therapies such as hyperbaric oxygen, IVIG, or flow-through HOCl are also lacking (11). Additionally, further investigation in reliable serologic biomarkers capable of distinguishing PNF from orbital cellulitis or Sweet’s syndrome is warranted (24).
